# Supplementary material for: Robust design of LAMP assays for in-field detection of major bacterial vascular diseases of banana
Source: PLoS One. 2026 Jul 16;21(7):e0337387. doi: 10.1371/journal.pone.0337387 (PMC13375027; doi:10.1371/journal.pone.0337387)
Supplement: S1 Fig — (A) Uganda, (B) Indonesia (Java), (C) Ecuador, and (D) Jamaica. The numbers of samples collected and tested by LAMP are indicated in red. The map was generated using OpenStreetMap data (©OpenStreetMap contributors. https://www.openstreetmap.org). (DOCX) [file pone.0337387.s001.docx]

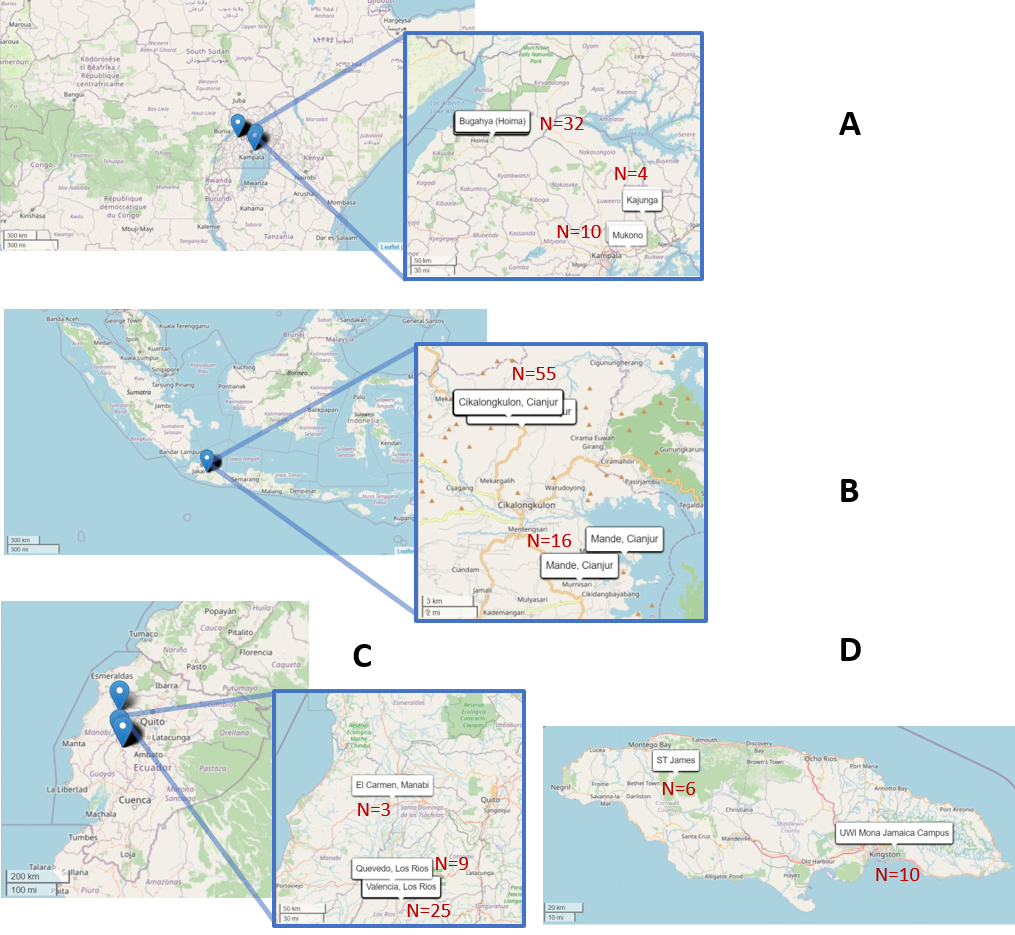


**Figure S1**. Areas where the field surveys were conducted: Uganda (**A**), Indonesia (Java) (**B**), Ecuador (**C**), and Jamaica (**D**). The numbers of samples collected and tested by LAMP are indicated in red. The map was generated using OpenStreetMap data (©OpenStreetMap contributors <https://www.openstreetmap.org>).
