# Supplementary material for: Robust design of LAMP assays for in-field detection of major bacterial vascular diseases of banana
Source: PLoS One. 2026 Jul 16;21(7):e0337387. doi: 10.1371/journal.pone.0337387 (PMC13375027; doi:10.1371/journal.pone.0337387)
Supplement: S2 Fig — “D” followed by a number refers to the day after inoculation when the observation occurred. (DOCX) [file pone.0337387.s002.docx]

**
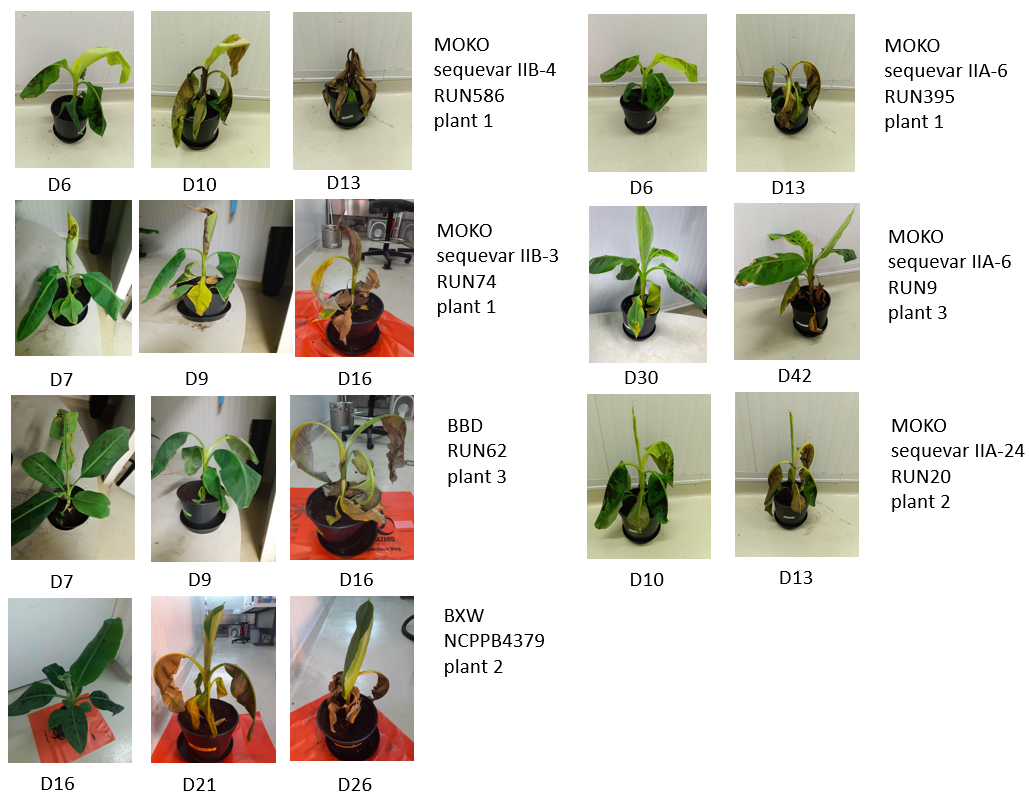
**

**Figure S2.** **Symptoms observed over time after inoculation of strains belonging to the species *R. solanacearum* (Moko disease), *R. syzygii* subsp. *celebesensis* (BBD), and *X. vasicola* pv*. musacearum* (BXW).** “D” followed by a number refers to the day after inoculation when the observation occurred.
