# Supplementary material for: Robust design of LAMP assays for in-field detection of major bacterial vascular diseases of banana
Source: PLoS One. 2026 Jul 16;21(7):e0337387. doi: 10.1371/journal.pone.0337387 (PMC13375027; doi:10.1371/journal.pone.0337387)
Supplement: S3 Fig — Tendency curves with their 95% confidence intervals were calculated using local polynomial regressions (locally estimated scatterplot smoothing [LOESS] method). (DOCX) [file pone.0337387.s003.docx]

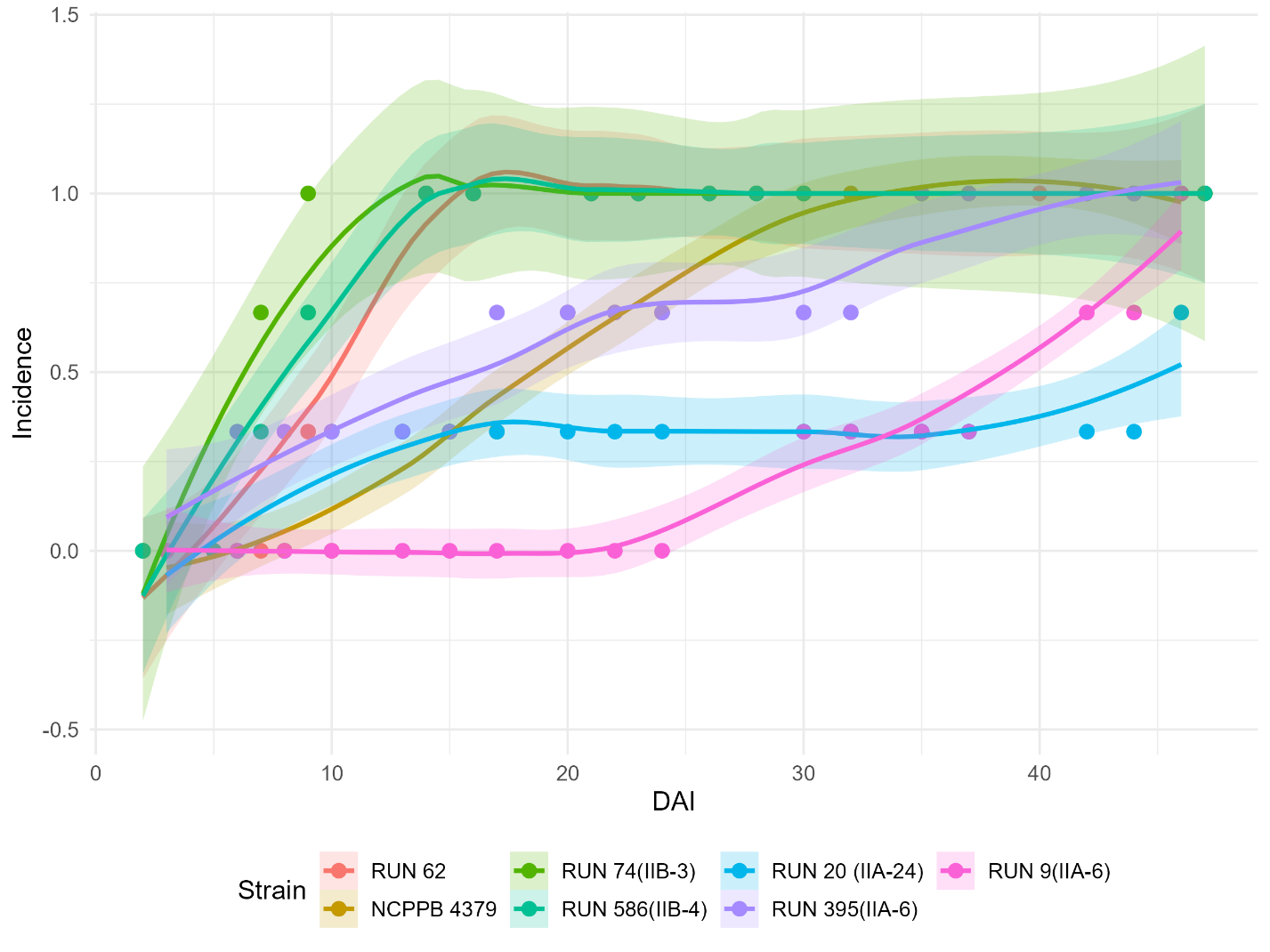


**Figure S3**. Percentage of diseased plants over time following inoculation with the different pathogens. Tendency curves with their 95% confidence intervals were calculated using local polynomial regressions (locally estimated scatterplot smoothing [LOESS] method).
