# Supplementary material for: Robust design of LAMP assays for in-field detection of major bacterial vascular diseases of banana
Source: PLoS One. 2026 Jul 16;21(7):e0337387. doi: 10.1371/journal.pone.0337387 (PMC13375027; doi:10.1371/journal.pone.0337387)
Supplement: S4 Fig — A. Each symbol corresponds to an observed LAMP TTR value. Symbol shapes represents the different banana tissues sampled; up-pointing triangles: pseudostem, upper part; down-pointing triangles: pseudostem, lower part; circles: corm. Symbol colors indicate the extraction method used: extraction kit (purple) or simplified NaOH-based extraction (green). B. Bacterial concentrations expressed in CFU/ml, recovered from the different tissues and plants sampled. (DOCX) [file pone.0337387.s004.docx]

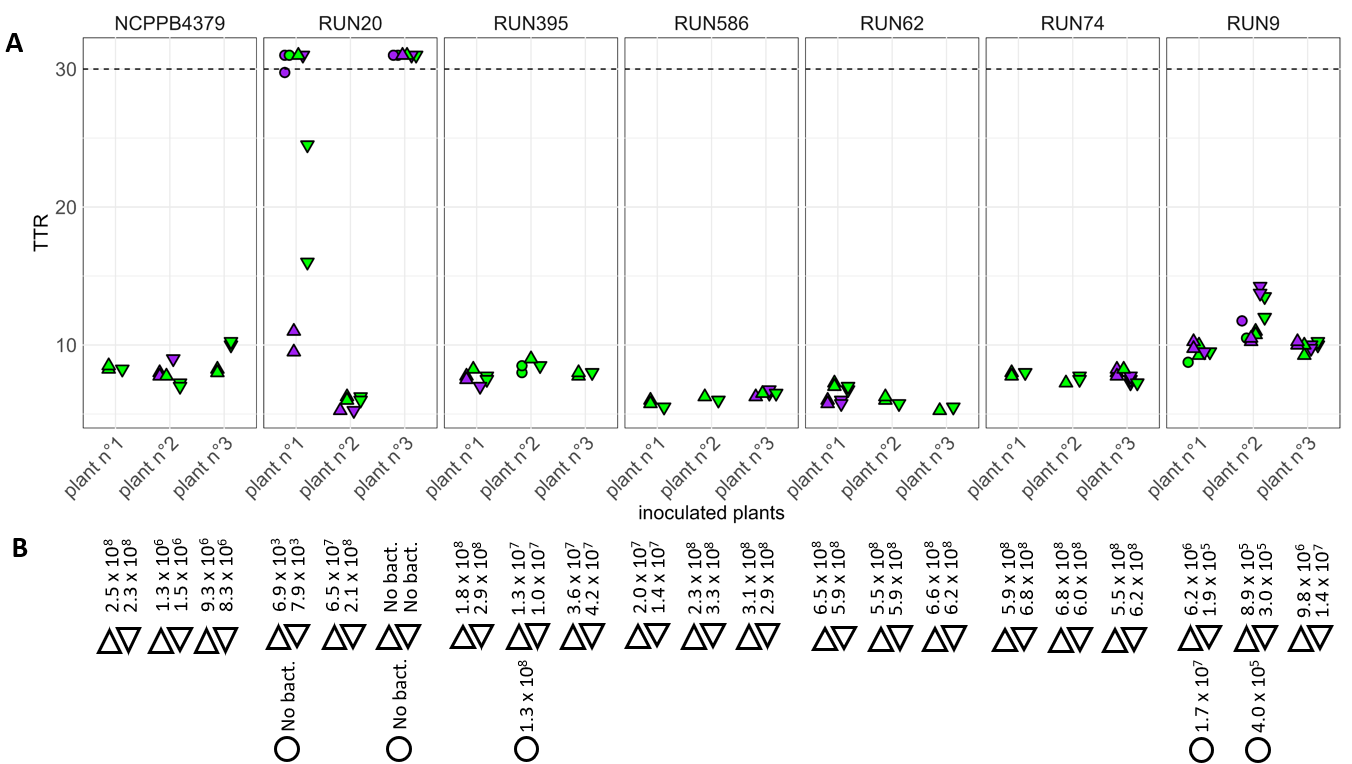


**Figure S4.** **LAMP signals (A) and bacterial concentrations (B) obtained from inoculated banana plants**. **A**. Each symbol corresponds to an observed LAMP TTR value. Symbol shapes represents the different banana tissues sampled; up-pointing triangles: pseudostem, upper part; down-pointing triangles: pseudostem, lower part; circles: corm. Symbol colors indicate the extraction method used: extraction kit (purple) or simplified NaOH-based extraction (green). **B**. Bacterial concentrations expressed in CFU/ml, recovered from the different tissues and plants sampled.
