# Supplementary material for: Robust design of LAMP assays for in-field detection of major bacterial vascular diseases of banana
Source: PLoS One. 2026 Jul 16;21(7):e0337387. doi: 10.1371/journal.pone.0337387 (PMC13375027; doi:10.1371/journal.pone.0337387)
Supplement: S5 Fig — The tree was constructed with RAxML as implemented in Gubbins v3.3.3, based on 44,522 nonrecombinant single nucleotide polymorphisms (SNPs) obtained from whole-genome alignment using Parsnp v1.7.4. Branch support values were calculated from 1,000 bootstrap replicates. The tree scale indicates the number of substitutions per genome. (DOCX) [file pone.0337387.s005.docx]

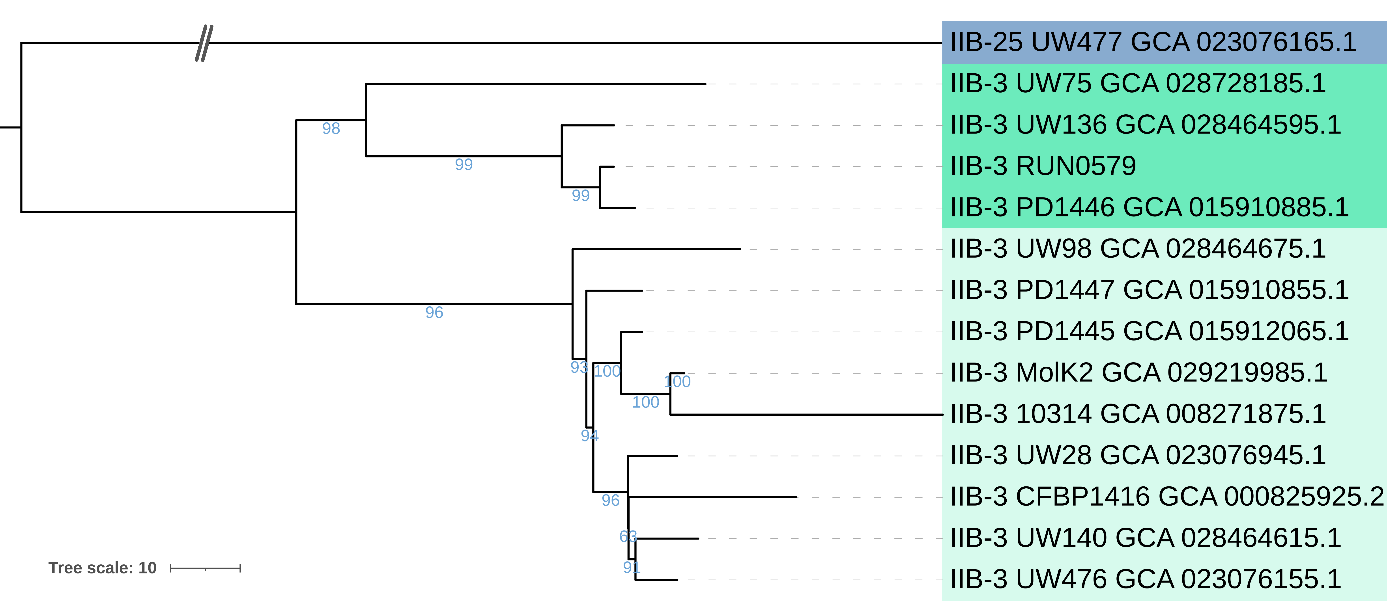


**Figure S5.** Phylogenetic tree of *Ralstonia solanacearum* sequevar IIB-3 strains, using a sequevar IIB-25 strain as outgroup. The tree was constructed with RAxML as implemented in Gubbins v3.3.3, based on 44,522 nonrecombinant single nucleotide polymorphisms (SNPs) obtained from whole-genome alignment using Parsnp v1.7.4. Branch support values were calculated from 1,000 bootstrap replicates. The tree scale indicates the number of substitutions per genome.
